# Early diagnosis and treatment of acute brucellosis knee arthritis complicated by acute osteomyelitis: two cases report

**DOI:** 10.1186/s12879-022-07392-5

**Published:** 2022-05-04

**Authors:** Jie Wang, Qiang Zhang

**Affiliations:** Department of Orthopedics, Beijing Ditan Hospital, Capital Medical University, Beijing, 100015 China

**Keywords:** Brucellosis, Septic knee arthritis, Acute osteomyelitis, Synovitis

## Abstract

**Background:**

Brucellosis is an endemic systemic infectious disease, the most common complication is bone and joint involvement. Sacroiliac joint and spinal joint are the most frequently involved sites in adults, but knee joint infection is rare, and acute infectious knee arthritis complicated by acute osteomyelitis is even extremely uncommon in adults. Here, we report two cases of acute septic knee arthritis complicated by acute osteomyelitis caused by *Brucella melitensis* (*B. melitensis*).

**Case presentation:**

Both patients had a history of traveling in animal husbandry areas within three months. On clinical examination, their right knee joint was tender, swollen, had limited movement and an effusion was present. Imaging examination showed effusion and synovial thickening of the right knee joint, as well as subchondral bone edema of the distal femur and proximal tibia. Laboratory examination showed that the serum agglutination test (SAT) in both patients were positive (1: 640 and 1: 320) without leukocytosis, although the proportion of lymphocytes, erythrocyte sedimentation rate (ESR) and C-reactive protein (CRP) significantly increased. Both patients underwent knee joint aspiration. Real-time polymerase chain reaction (Real-time PCR) analysis of synovial fluid showed that there was *B. melitensis*, and blood bacterial culture was negative. We determined that two patients had acute brucellosis knee arthritis complicated by acute osteomyelitis. Antibiotic treatment was given during hospitalization consisting of doxycycline (0.1 g po bid) and rifampicin (0.6 g po qd) for six weeks, and the changes of inflammatory indexes were closely monitored. At discharge, the symptoms had completely resolved, imaging abnormalities disappeared, and inflammatory indexes returned to normal. There was no recurrence of the disease at 1-year follow-up.

**Conclusion:**

Acute brucellosis knee arthritis complicated by acute osteomyelitis is a rare but serious complication of brucellosis in adults. There is no obvious specificity of clinical manifestation and imaging examination. Early diagnosis and treatment can prevent the occurrence of knee joint deformity and even pathological fracture. Clinicians should fully consider the possibility of brucellosis where the travel or occupational history is suggestive.

## Background

More than 500,000 people in the world are infected with *Brucella* every year [1]. The incidence of brucellosis in China is increasing, and the annual average incidence is 87.2/100,000 [2, 3]. Brucellosis is an endemic zoonotic disease, and the species that infect humans is usually *B. melitensis* [2]. Complications of bone and joint involvement occur in patients of 10–85%. Sacroiliac joint and spinal joint are the most common sites involved, but knee joint and bone marrow involvement are rare in adults [4]. Compared with other types of septic knee arthritis or osteomyelitis, the clinical manifestations and imaging examination of brucellosis knee arthritis or osteomyelitis are non-specific, with consequent misdiagnosis and suboptimal therapy leading to serious complications [5]. At present, the diagnosis of brucellosis mainly depends on the SAT, bacterial culture, enzyme-linked immunosorbent assay (ELISA), Real-time PCR, Gram stain, modified Ziehl–Neelsen stain, and Giemsa stain [6–9]. Bacteria and toxins play a major role in the acute phase, while delayed allergic reaction and the formation of granuloma are the main ones in the chronic phase [10].

## Case presentation

### Case 1

**Fig. 1:**
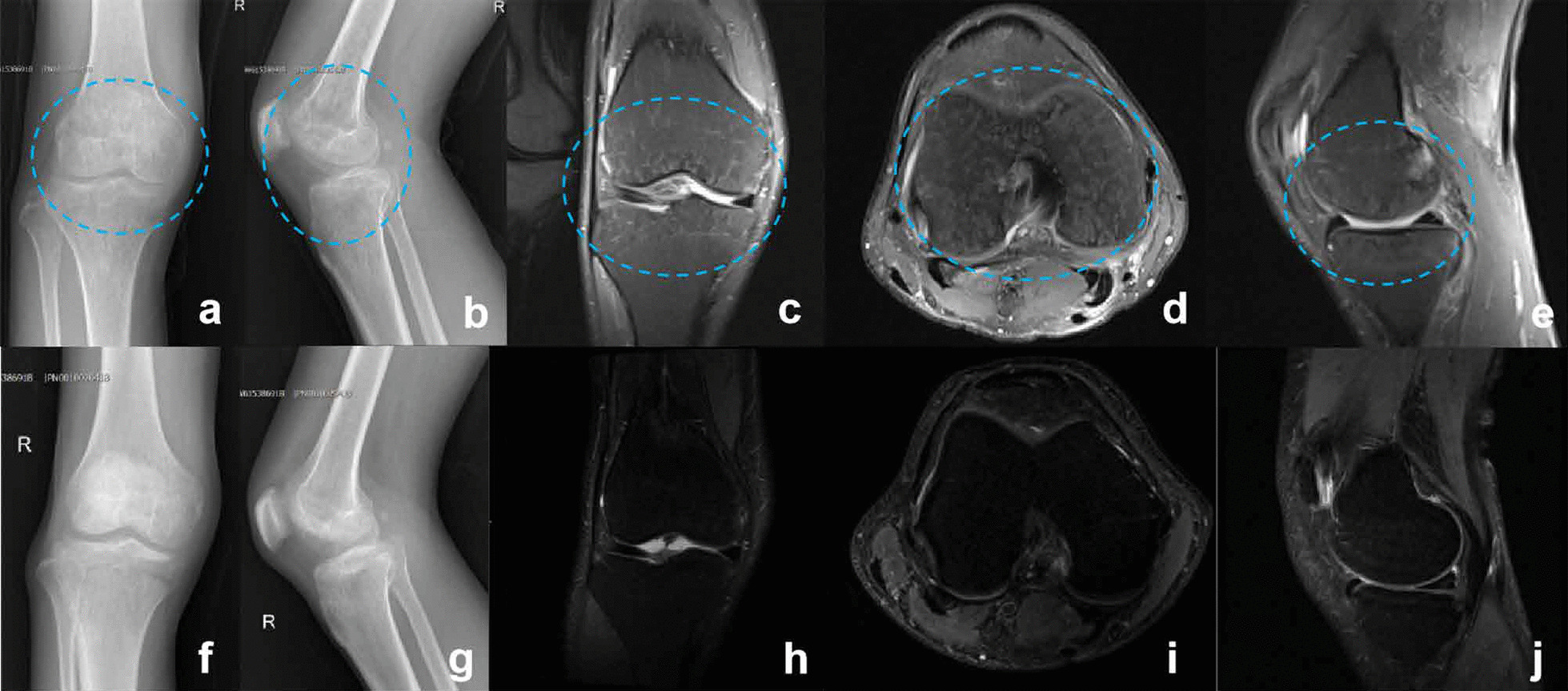
Patient 1: X-ray images showed multiple cystic low density areas (blue circle) of distal right femur and proximal tibia (**a**, **b**). PDWI sequence of MRI showed effusion and synovial thickening of the right knee joint, and subchondral bone marrow edema (blue circle) of the distal femur and proximal tibia (**c**–**e**). Post-treatment X-ray images showed the low density area was significantly reduced (**f**, **g**). Post-treatment PDWI sequence of magnetic resonance images indicates the high signal area of bone marrow edema had disappeared (**h**–**j**)

**Fig. 2:**
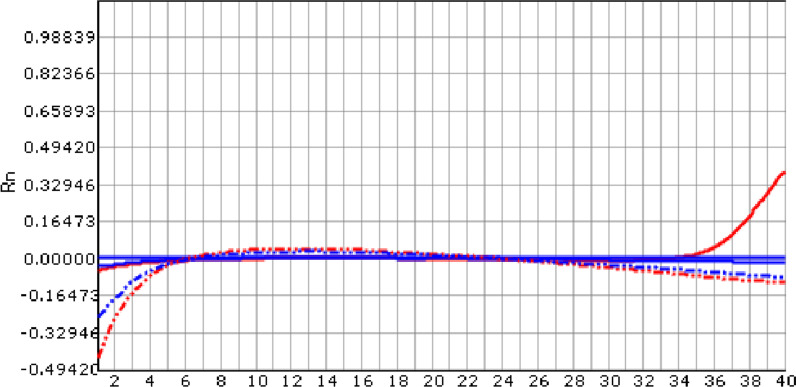
Patient 1: The Real-time PCR of purulent joint synovial fluid of right knee showed that there was *Brucella melitensis* in the synovial fluid, DNA content of *Brucella melitensis* (Solid red line) increased in 35 cycles

A 43-year-old Chinese man developed fever with pain and limitation of movement of the right knee joint less than three months duration without obvious cause. Knee joint aspiration was negative and antibiotic treatment was started in the local hospital; however, symptoms were not significantly improved and fever persisted up to 38.1 °C. The patient attended the orthopaedic clinic of our hospital for further assessment. Medical history revealed that he traveled to an animal husbandry area (Inner Mongolia) in china about 3 months prior and ate undercooked mutton, the specific date is July 16th, 2019. We suspected that the patient had been infected with *Brucella* by eating contaminated mutton. Physical examination showed that the right knee joint was obviously swellen, the distal femur and proximal tibia had significant bony tenderness, flexion and extension of the knee joint was limited, and right patellar tap sign was positive. Blood tests showed that the white blood cell count was normal (6.8 × 10^9^/L), the proportion of lymphocytes were increased, ESR and CRP levels were 135 mm/h and 52 mg/L respectively, procalcitonin (PCT) level of 0.14 ng/ml, antinuclear antibody were negative, anti-streptolysin O (ASO) and rheumatoid factor were all in the normal range, and the result of SAT was positive (1: 640). Anteroposterior and lateral X-ray of the right knee joint showed multiple subchondral low-density areas in the distal right femur and proximal tibia (Fig. 1a, b). Proton density weighted image (PDWI) sequence of magnetic resonance imaging (MRI) showed effusion and synovial thickening of the right knee joint, and subchondral bone marrow edema of the distal femur and proximal tibia (Fig. 1c–e). The blood bacterial culture was negative. Purulent joint synovial fluid of right knee was extracted by joint aspiration and sent to the laboratory for Real-time PCR examination, the results showed that there was *B. melitensis* in the synovial fluid (Fig. 2). We concluded that the patient had acute brucellosis knee arthritis complicated by acute osteomyelitis and was treated with doxycycline (0.1 g po bid) and rifampicin (0.6 g po qd) for six weeks during hospitalization. At discharge, the swelling of the right knee had resoved and there was no pain or tenderness of the right knee, distal femur or proximal tibia. Repeat X-ray and MRI of the right knee joint showed that the low density area on the X-ray was significantly reduced, and the high signal area of bone marrow edema on the PDWI sequence of MRI had disappeared (Fig. 1f–j). White blood cell count was normal (6.4 × 10^9^/L), proportion of lymphocytes was normal, ESR and CRP levels were 15 mm/h and 8 mg/L respectively, and the titer of SAT turned to 1: 80. There was no recurrence of the disease at 1-year follow-up.

### Case 2

**Fig. 3:**
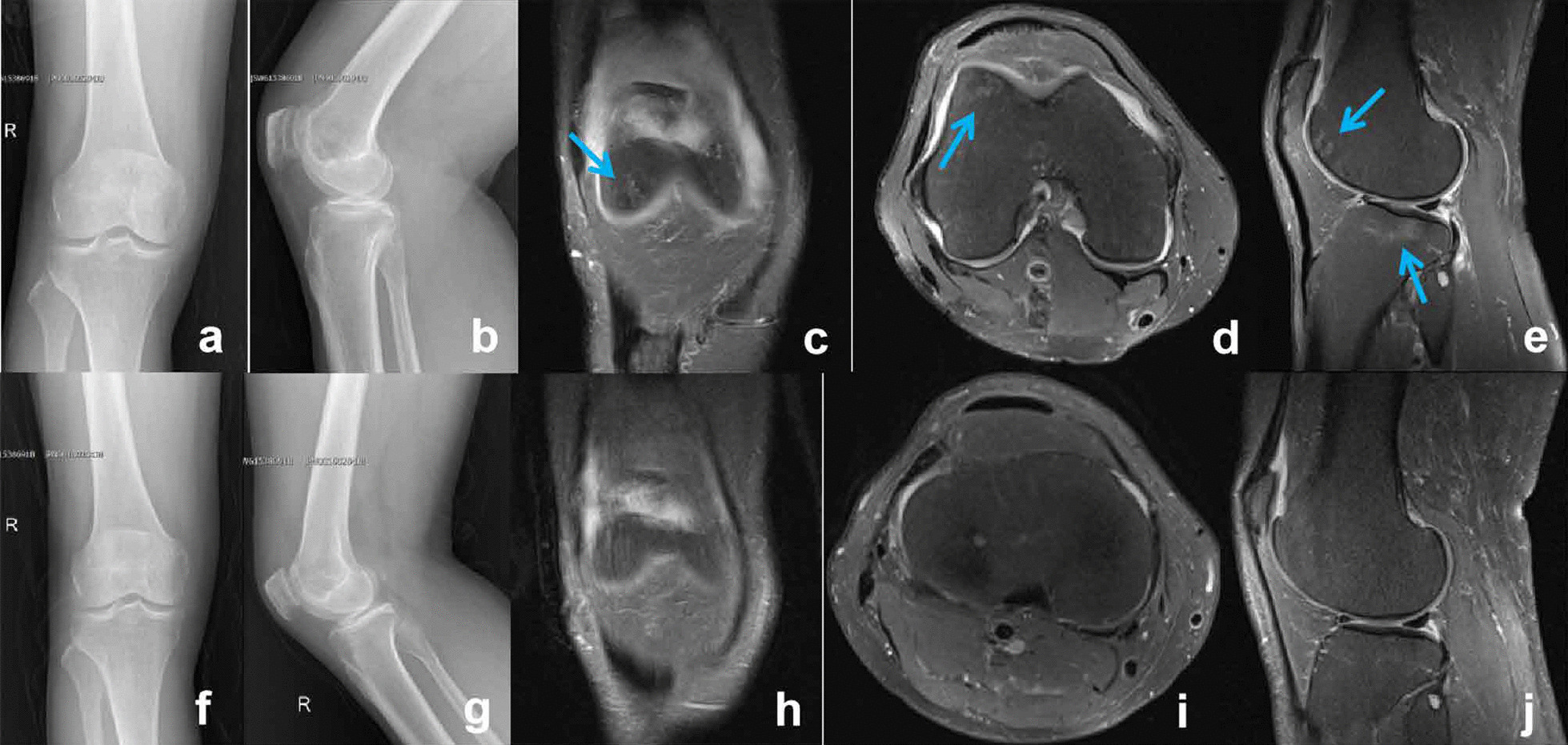
Patient 2: X-ray showed no obvious abnormality (**a**, **b**). PDWI sequence of MRI showed effusion, synovitis and bone marrow edema (blue arrow) deep to the surface of the right femoral trochlea and tibial plateau (**c**–**e**). Post-treatment X-ray images showed no obvious abnormality (**f**, **g**). Post-treatment PDWI sequence of magnetic resonance images indicates the edema of bone marrow disappeared and the effusion in articular cavity decreased significantly (**h**–**j**)

**Fig. 4:**
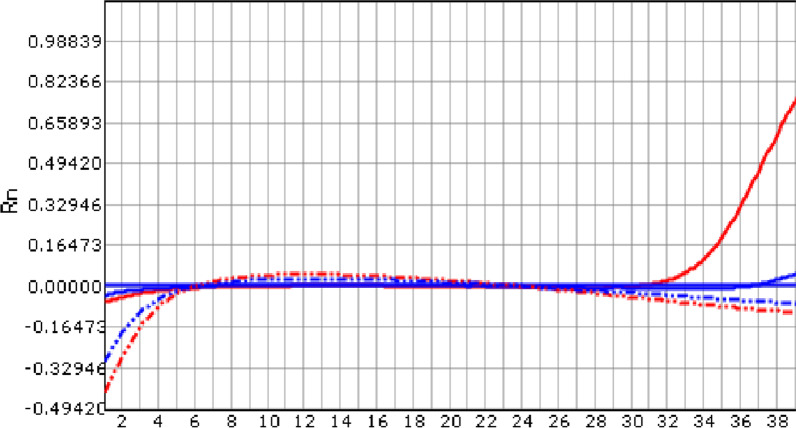
Patient 2: The Real-time PCR of purulent synovial fluid of right knee joint showed that there was *Brucella melitensis*, DNA content of *Brucella melitensis* (Solid red line) increased in 31 cycles

A 38-year-old Chinese man developed profuse sweating with a fever of up to 37.9 °C of two months without apparent cause. A month ago, he developed pain and swelling of the right knee joint. He was treated in a local hospital with cephalosporin antibiotics, however his fever persisted and right knee joint pain gradually worsened. The patient attended the orthopaedic clinic of our hospital for further diagnosis and treatment. Medical history showed that he had handled sheep with hands that had broken skin in Qinghai Province two months ago (August 13th, 2018), and we suspected that the patient might have contracted brucellosis through direct contact. On admission, the right knee joint was slightly swollen, the right distal femur and proximal tibia were tender, active and passive flexion of the right knee joint was limited, and right patellar tap sign was positive. Blood tests showed a mild increase in white blood cell count (10.4 × 10^9^/L), an increase in the proportion of lymphocytes, ESR and CRP levels of 153 mm/h and 46 mg/L respectively, PCT level of 0.17 ng/ml, negative antinuclear antibody, no abnormality of ASO and rheumatoid factor, and positive results of SAT (1: 320). There was no obvious abnormality seen on anteroposterior and lateral X-ray (Fig. 3a, b). PDWI sequence of MRI showed effusion, synovitis and bone marrow edema deep to the surface of the right femoral trochlea and tibial plateau (Fig. 3c–e). Bacterial culture of blood was negative. Aspiration of the right knee joint yielded purulent synovial fluid and Real-time PCR showed that there was *B. melitensis* in the synovial fluid (Fig. 4). We concluded that the patient had developed acute brucellosis right knee arthritis complicated by acute osteomyelitis, he take doxycycline (0.1 g po bid) and rifampicin (0.6 g po qd) for six weeks during hospitalization. At discharge, flexion and extension of the right knee was unrestricted, and there was no obvious abnormality seen on X-ray or MRI of the right knee (Fig. 3f–j). White blood cell count was normal (8.6 × 10^9^/L), the proportion of lymphocytes was normal, the ESR and CRP levels were 12 mm/h and 6 mg/L respectively, and the titer of SAT turned to 1: 40. There was no recurrence of the disease at 1-year follow-up.

## Discussion and conclusions

The type of bone and joint involvement in brucellosis mainly depends on the age of the patient, with sacroiliac joint and spinal disease predominating in adults, and knee and ankle joint infection in children and minors [11]. Acute septic knee arthritis and acute osteomyelitis are two different types of disease. There is no report of acute brucellosis knee arthritis complicated by acute osteomyelitis of adult in Chinese or world literature at present. People contract *Brucella* mainly through occupational contact (e.g. veterinary, slaughtering, animal husbandry) and consumption of unpasteurized milk and milk products via the digestive tract [2]. In this report, both patients had traveled to the areas endemic with brucellosis. The second patient handled sheep with hands that had broken skin; while the first patient only ate the undercooked mutton, the possibility of aerosol transmission also exists [12]. Acute osteomyelitis is mainly caused by hematogenous infection in adults, rarely through local spread of infection [13]. The blood bacterial culture of the two patients in this report were negative and the hemogram infection index were not high. We inferred that acute osteomyelitis was mainly caused by local spread infection of acute brucellosis knee arthritis.

The MRI findings of brucellosis knee arthritis is mainly of effusion and synovial thickening, without specificity. The specific findings of acute brucellosis osteomyelitis are multiple patchy edema of bone marrow on MRI and multiple focal low-density lesions on X-ray. Early diagnosis and treatment can reverse the abnormalities of clinical manifestations and imaging of acute osteomyelitis. Delayed diagnosis and treatment of acute osteomyelitis can progress to chronic osteomyelitis, significantly increasing the risk of pathological fractures [14, 15].

The most common diagnostic method of brucellosis is by serologyical methods, including SAT. A titer of SAT ≥ 1: 160 is highly suggestive of brucellosis[16]. Real-time PCR is an efficient detection method in detecting the etiology of bacteremia or local infection, it can also exclude chronic brucellosis [17]. The gold standard of diagnosis is blood culture or tissue culture (synovial fluid or bone marrow), but the isolation of microorganisms is very difficult, bone marrow puncture is an invasive examination, which many patients cannot accept [18].

If laboratory technicians do not take proper precautions when performing bacterial culture operations, they may become infected with *Brucella* through damaged skin or respiratory tracts [19, 20]. Microbiology laboratories must take necessary safety measures to provide triple protection to laboratory technicians, the experimental environment, and the experimental samples. Laboratory technicians can reduce the incidence of *Brucella* infection by reducing operational errors like needle inoculations, culture spills, and glass fragments injuries [21]. The living bacteria isolation, culture centrifugation, and freezing of *Brucella* should be performed in Biosafety Level 3 (BSL-3) facility [22]. It is straightforward for the *Brucella* to infect the respiratory system and mucosal system by aerosols; therefore, dangerous methods such as sniffing out the smell of bacteria that can cause brucellosis should be abandoned when dealing with suspicious cultures [19, 20, 23]. *Brucella* should be inspected by personnel wearing masks, and the procedure should be conducted within a biosafety cabinet to avoid the production of aerosols from strain isolation and culture operations [24, 25]. In patients with brucellosis, *Brucella* can be isolated from blood, bone marrow, synovial fluid, cerebrospinal fluid, and urine; the positive culture rate in acute stages and prior to antibiotic treatment is higher than that in chronic stages; while the positive rate of Real-time PCR (Real-time PCR) is higher than bacterial culture, it can also identify specific *Brucella* species [26].

The recommended drug treatment for brucellosis includes a combination of two or three antibiotics. These are prescribed according to whether the disease is complex or not. The appropriate combination of antibiotics should be selected according to the patient's condition. The triple therapy recommended by the World Health Organization (WHO), doxycycline (0.1 g bid), rifampicin (0.6 g qd) and streptomycin (1 g qd) may be most effective if given within 6 months of disease onset [27]. In the case of recurrence, patients should receive a standard drug regime consisting of doxycycline combined with streptomycin or rifampicin for six weeks, which can be appropriately prolonged when there is compex bone and joint infection [28].

Acute brucellosis knee arthritis complicated by acute osteomyelitis is a rare but serious complication of brucellosis in adults. There are no obvious specific features on clinical and imaging examination. Early diagnosis and treatment can prevent the occurrence of knee joint deformity or pathological fracture. Clinicians should consider brucellosis where the travel or occupational history is suggestive.

## Data Availability

All data and materials are available with the first author.

## Abbreviations

*B. Melitensis*: *Brucella melitensis*
SAT: Serum agglutination test
ESR: Erythrocyte sedimentation rate
CRP: C-reactive protein
Real-time PCR: Real-time polymerase chain reaction
ELISA: Enzyme-linked immunosorbent assay
PCT: Procalcitonin
ASO: Anti-streptolysin O
PDWI: Proton density weighted image
MRI: Magnetic resonance imaging
BSL-3: Biosafety Level 3
WHO: World Health Organization
